# Diagnostic efficiency among Eu-/C-/ACR-TIRADS and S-Detect for thyroid nodules: a systematic review and network meta-analysis

**DOI:** 10.3389/fendo.2023.1227339

**Published:** 2023-08-31

**Authors:** Longtao Yang, Cong Li, Zhe Chen, Shaqi He, Zhiyuan Wang, Jun Liu

**Affiliations:** ^1^ Department of Radiology, The Second Xiangya Hospital, Central South University, Changsha, China; ^2^ Department of Thoracic Surgery, The Second Xiangya Hospital, Central South University, Changsha, China; ^3^ Department of Ultrasound, The Affiliated Cancer Hospital of Xiangya School of Medicine, Central South University, Changsha, Hunan, China; ^4^ Clinical Research Center for Medical Imaging in Hunan Province, Changsha, China; ^5^ Department of Radiology Quality Control Center in Hunan Province, Changsha, China

**Keywords:** thyroid imaging reporting and data system, Eu-TIRADS, ACR TIRADS, C-TIRADS, S-Detect, diagnostic performance, network meta-analysis

## Abstract

**Background:**

The performance in evaluating thyroid nodules on ultrasound varies across different risk stratification systems, leading to inconsistency and uncertainty regarding diagnostic sensitivity, specificity, and accuracy.

**Objective:**

Comparing diagnostic performance of detecting thyroid cancer among distinct ultrasound risk stratification systems proposed in the last five years.

**Evidence acquisition:**

Systematic search was conducted on PubMed, EMBASE, and Web of Science databases to find relevant research up to December 8, 2022, whose study contents contained elucidation of diagnostic performance of any one of the above ultrasound risk stratification systems (European Thyroid Imaging Reporting and Data System[Eu-TIRADS]; American College of Radiology TIRADS [ACR TIRADS]; Chinese version of TIRADS [C-TIRADS]; Computer-aided diagnosis system based on deep learning [S-Detect]). Based on golden diagnostic standard in histopathology and cytology, single meta-analysis was performed to obtain the optimal cut-off value for each system, and then network meta-analysis was conducted on the best risk stratification category in each system.

**Evidence synthesis:**

This network meta-analysis included 88 studies with a total of 59,304 nodules. The most accurate risk category thresholds were TR5 for Eu-TIRADS, TR5 for ACR TIRADS, TR4b and above for C-TIRADS, and possible malignancy for S-Detect. At the best thresholds, sensitivity of these systems ranged from 68% to 82% and specificity ranged from 71% to 81%. It identified the highest sensitivity for C-TIRADS TR4b and the highest specificity for ACR TIRADS TR5. However, sensitivity for ACR TIRADS TR5 was the lowest. The diagnostic odds ratio (DOR) and area under curve (AUC) were ranked first in C-TIRADS.

**Conclusion:**

Among four ultrasound risk stratification options, this systemic review preliminarily proved that C-TIRADS possessed favorable diagnostic performance for thyroid nodules.

**Systematic review registration:**

https://www.crd.york.ac.uk/prospero, CRD42022382818.

## Introduction

Thyroid nodule is a common occurrence in clinical practice. Ultrasonography plays an irreplaceable role in the early detection of thyroid cancer with merits of being noninvasive, convenient, and affordable (1). As high-resolution ultrasound technology and the standard of living improve, the detection rate of thyroid nodules has increased gradually. Ultrasound examination can detect 30% to 67% of thyroid nodules, with malignant nodules accounting for 7% to 15% (2). In most cases, due to possibly overlapped imaging phenotypes between benign and malignant thyroid nodules, the interpretation of ultrasound images still relies on the subjective discrimination of radiologists (3). Thus, it cannot accurately determine the malignancy of thyroid tumors via dependence on single imaging features, which calls for making elaborate evaluation criteria (4). To address this, TIRADS was proposed in 2009, which was inspired by the Breast Imaging Reporting and Data System (BIRADS) of the ACR (5). Subsequently, numerous classifying systems suitable for various countries gradually progressed in later periods, whose versions released since 2017 encompassed Eu-TIRADS, ACR TIRADS, and C-TIRADS (6). Hence, application of TIRADS in worldwide ultrasound departments is relatively disorganized, which causes challenges for doctors in underdeveloped countries to choose the optimal diagnostic system (6). Moreover, advancement in medical artificial intelligence facilitates the establishment of computer-aided diagnosis (CAD) systems, whose classifying effectiveness has been initially verified in the examination of breast and thyroid nodules (7). S-Detect, a widely applied ultrasound CAD, is employed for recognizing thyroid nodules through early data learning and algorithm optimization (8). Although the Expert Committee agrees that each risk stratification option including TIRASD and CAD has its own advantages and disadvantages, there is a necessity to propose the most universal and efficient system for better clinical service (9).

Previous systematic reviews explored the diagnostic performance of various ultrasound risk stratification systems for thyroid nodules (10, 11), but traditional meta-analysis methods were not ideal for indirectly comparing multiple diagnostic techniques from separate studies. Network meta-analysis is a method that allows for indirect and direct comparison of multiple treatment or testing options by parsing data extracted from different studies to rank these regimens (12). Therefore, this systemic review carried out a comprehensive quantitative synthesis of discovered studies by using network meta-analysis to clarify clinical diagnostic performance among the latest risk stratification systems since 2017 and S-Detect in distinguishing benign from malignant thyroid nodules.

## Evidence acquisition

### Search strategy and study selection

This network meta-analysis study was executed in accordance with the Preferred Reporting Items for Systematic Reviews and Meta-Analyses (PRISMA) guidelines. The study protocol was registered with PROSPERO (york.ac.uk) and assigned registration number CRD42022382818. A comprehensive literature search was performed by using the Population, Intervention, Comparison, and Outcome (PICO) format to answer the following questions: How to compare the diagnostic performance among ACR TIRADS, Eu-TIRADS, S-DETECT, and C-TIRADS by taking cytologic or histopathological tests as reference standards in patients who underwent ultrasound detection of thyroid nodules?

PubMed, EMBASE, and Web of Science databases were explored from the date of database creation to December 8, 2022, complying with the following search terms: ACR TIRADS, EU-TIRADS, C-TIRADS, S-detect, qualitative diagnosis, and thyroid nodule. The search strategy was mapped out by a researcher with six months of information retrieval experience. Two researchers independently screened the search results, and any discrepancies were resolved through discussion with a third investigator. After removing duplicates and non-research articles, such as reviews, conference reports, and case reports, as well as excluding articles that did not meet research question criteria, a final sample of studies was obtained. These studies provided sufficient data to assess the diagnostic performance of at least one of the four ultrasound risk stratification options.

### Data extraction

Two investigators were responsible for data extraction, and any conflicting results were decided through discussion with a third investigator. Recorded information covered relevant demographic factors such as country, ethnicity, sex ratio, mean age, the number of patients and nodules, as well as the mean diameter of benign and malignant nodules. Additionally, ultrasound risk stratification methods, optimal cut-off values, and reference criteria in each study were noted. The investigators also extracted the number of true-positive, true-negative, false-positive, and false-negative nodules based on the reference criteria to calculate diagnostic performance of each risk stratification system.

In 2017, the TIRADS Committee of the ACR published a white paper introducing a new risk stratification format for thyroid nodules, known as ACR TIRADS. This system categorizes nodules as benign (TR1, 0 points), not suspicious (TR2, 2 points), mildly suspicious (TR3, 3 points), moderately suspicious (TR4, 4-6 points), or highly suspected malignant (TR5, 7 points) (13). Eu-TIRADS identifies thyroid nodules as benign (TR2), low risk (TR3), intermediate risk (TR4) or high risk (TR5) (14). C-TIRADS categorizes thyroid nodules as non-nodules (C-TIRADS TR1), benign (C-TIRADS TR2), potentially benign (C-TIRADS TR3), or varying levels of malignancy suspicion (C-TIRADS TR4a-c, TR5), with TR5 nodules having the highest malignant probability (>90%) (15). In addition to these options, S-Detect, a real-time CAD system software, is integrated into ultrasound systems that can automatically identify the region of interest associated with nodule, thereby calculating contour and quantifying various ultrasound manifestations of the nodule, including size, composition, shape, orientation, echogenicity, and spongiform. These quantitative features are then analyzed by the AI algorithm to diagnose whether the nodule is benign or malignant (16).

### Quality assessment

The risk of bias and applicability concerns of the included studies were assessed based on QUADAS-2 tool, which was routinely employed for analyzing validation studies of diagnostic criteria. Each item was rated as low, high, or unclear risk. This tool estimates the risk of bias across four domains: patient selection, index test, reference standard, as well as flow and timing. Additionally, it assesses applicability concerns, focusing on the first three domains. Two independent reviewers (C.L. and S.H.) commented on included studies, with any disagreement addressed by consensus. The QUADAS-2 scale was then completed based on commentary information.

### Statistical analysis

The best sensitivity, specificity, and accuracy of each stratification option were determined using Stata13.1 “midas” packages, based on the number of true-positive, false positives, true-negative, and false-negative assessments extracted from the individual studies. I^2^ was computed to quantify the heterogeneity among inter-studies in terms of sensitivity, specificity, and accuracy for each system, and was considered to be substantial when at least 75%. Then, through included one-arm/two-arm/three-arm studies, direct and indirect comparisons of performance metrics (e.g., sensitivity, specificity, et al.) among four options were performed through random effect network meta-analysis within the frequentist framework, based on risk category with the highest diagnostic accuracy in each option. Next, within-group heterogeneity and between-group heterogeneity were calculated. Finally, the risk stratification system evaluated by included medium-quantity studies was identified as reference option, and odds ratios (ORs) and 95% confidence intervals (CIs) were used to express the sensitivity, specificity, and accuracy with respect to that system for the remaining three systems.

This network meta-analysis was conducted by R package “rstan” (version 4.1.3; R Foundation for Statistical Computing, Vienna, Austria). Analysis of Variance (ANOVA) model based on Bayesian algorithm was applied to exhibit network meta-analysis among four systems by utilizing two independent binomial distributions to describe the true positive and true negative rates between benign and malignant thyroid nodules and meantime considering the correlation between sensitivity and specificity (17). There were 4 trials to be evaluated and 88 included studies. In certain study i, (Y_i1k_, Y_i2k_) referred to true positive and true negative, respectively; (N_i1k_, N_i2k_) referred to malignant and benign thyroid nodules, respectively; and (π_i1k_, π_i2k_) referred to “unobserved” sensitivity and specificity, respectively. The binomial distribution describing true positive and true negative between malignant and benign thyroid nodules was as follows:


$${\text{Y}}_{\text{ijk}}|{\text{π}}_{\text{ijk}},{\text{x}}_{\text{i}}∼\text{bin }\left({\text{π}}_{\text{ijk}},{\text{N}}_{\text{i}2\text{k}}\right),$$

I = 1,… I; j = 1, 2, k = 1,… K; x_i_ refers to covariate affecting π_ijk_.

Monte Carlo chain number, pre-iterations number, iteration number and step size were set at 3, 10000, 1000, and 5, respectively. Subsequently, absolute effect sizes including sensitivity and specificity, as well as relative effect sizes including risk ratio (RR), OR, and DOR were calculated.

Besides, we employed various statistical packages in R4.1.3 to analyze extracted data. Specifically, we utilized “mada” and “reshape” packages to conduct the Summary Receiver Operating Characteristic (SROC) analysis of four optimal categories from risk stratification systems. The forest plots respectively related to ORs of sensitivity and specificity were created using “forestplot” package. To visualize publication bias, we used the “meta” and “metafor” packages.

## Evidence synthesis

### Study selection

**Figure 1:**
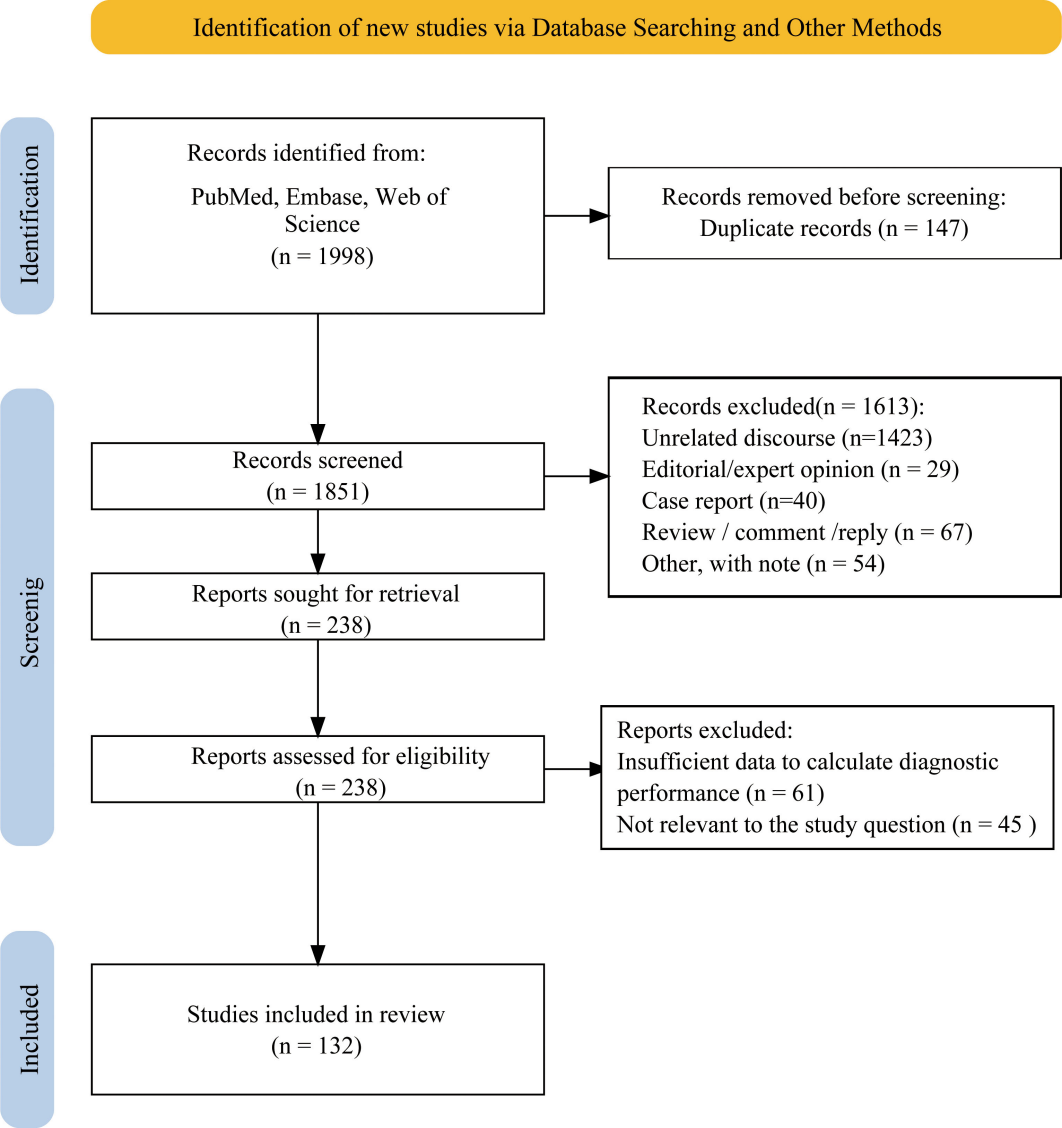
Study selection process. A total of 132 studies were included in this meta-analysis.

A total of 1998 articles were initially identified through multiple database searches, of which 147 duplicate articles were removed, leaving 1,851 articles for further review. After excluding 1,613 articles based on title and abstract screening, 238 articles were reviewed in full, where 61 articles lacked sufficient data to determine diagnostic performance, and 45 articles were irrelevant to the research topic. Ultimately, 132 studies were included in meta-analysis, comprising 90,451 nodules. **Figure 1** showed screening process. Specifically, 76 studies only analyzed ACR TIRADS, 10 studies only analyzed Eu-TIRADS, 5 studies only analyzed C-TIRADS and 5 studies only analyzed S-Detect. Furthermore, there were 21, 5, 2, and 4 studies comparing diagnostic effectiveness of ACR TIRADS and Eu-TIRADS, ACR TIRADS and C-TIRADS, Eu-TIRADS and S-Detect, as well as ACR TIRADS and S-Detect, respectively. In addition, only 3 studies examined the diagnostic performance of all three diagnostic systems including ACR-TIRADS, Eu-TIRADS, and C-TIRADS, and only 1 study examined ACR TIRADS, Eu-TIRADS, and S-Detect.

### Quality assessment

A summary of the risk of bias and applicability concerns for each domain was exhibited in **Supplementary Figure 1**. **Table 1** presented a detailed assessment for each domain. Most studies showed a low or unclear risk of bias in each domain. 33 enrolled studies had low risk of bias, while 14 studies were considered to have high risk of bias due to non-consecutive patient selection and inappropriate exclusions. The remaining studies with non-consecutive patient selection were considered to have an unclear risk of bias. In index test domain, most of studies had a low or unclear risk of bias because of the prespecified thresholds and blinding. The reference standard domain had a low risk of bias for most studies since they were based on cytological and pathological golden standards. In the flow and timing domain, 2 studies had high risk of bias due to introduced bias of patient flow, while the others were considered to have low or unclear risk of bias. In terms of applicability concerns, at high concern, there were 6 studies in one domain, 3 studies in two domains, and only 1 study in all domains, while the other studies had low or unclear concerns.

**Table 1:** Summary of risk stratification systems for evaluating ultrasound thyroid nodules in network meta-analysis.

Risk Stratification System	Approach	Risk Categories (Size Threshold for Biopsy at Category)
ACR TIRADS	Score based	•TR1, benign (no biopsy) •TR2, not suspicious (no biopsy) •TR3, mildly suspicious (≥25 mm; follow-up ultrasound if ≥15 mm) •TR4, moderately suspicious (≥15 mm; follow-up ultrasound if ≥10 mm) •TR5, highly suspicious (≥10 mm; follow-up ultrasound if ≥0.5 mm)
Eu-TIRADS	Pattern based	•TR2, benign (no biopsy) •TR3, low risk (≥20 mm) •TR4, intermediate risk (≥15 mm) •TR5, high risk (>10 mm; FNA or active surveillance if ≤10 mm)
C-TIRADS	Score based	Solid, microcalcifications, marked hypoechogenicity, indistinct border, irregular border or extrathyroidal extension, and vertical orientation are the ultrasonic characteristics of malignant nodules, while comet tail artifacts are the ultrasonic characteristics of benign nodules. Each malignant feature is given a score of 1 point, and if there are benign comet tail artifacts, the score is reduced by 1 point: •TR1, non-nodular •TR2, -1 score, benign •TR3, 0 score, potentially benign •TR4a, 1 score, low malignancy suspicion •TR4b, 2 score, moderate malignancy suspicion •TR4c, 3 or 4 score, extremely malignancy suspicion •TR5, 5 score, high malignancy
S-Detect	Pattern based	S-Detect focusing on six B-mode lexicons, i.e. orientation, margin, spongiform, composition, echogenicity, and shape, which recommend malignancy or benignancy of the lesion.

ACR TIRADS, American College of Radiology Thyroid Imaging Reporting and Data System; Eu-TIRADS, European Thyroid Association Thyroid Imaging Reporting and Data System; C-TIRADS, Chinese version of Thyroid Imaging Reporting and Data System; S-Detect, Computer aided diagnosis system using deep learning.

### Meta-analysis of individual risk stratification system

**Table 2** shows the diagnostic performance of the four stratification systems at each system’s risk category thresholds. 107 studies evaluated ACR TIRADS (18–124). After meta-analysis, in the best risk category threshold TR5 of ACR TIRADS, sensitivity ranged from 65% to 76%; specificity ranged from 84% to 89%; and accuracy ranged from 77% to 80%. 37 studies evaluated Eu-TIRADS (20, 22, 28, 48, 55, 65, 66, 70, 73, 74, 78, 80–82, 85, 86, 92, 93, 95, 99–101, 115, 118, 121, 125–136). After meta-analysis, in the best risk category threshold TR5 of Eu-TIRADS, sensitivity ranged from 66% to 82%; specificity ranged from 75% to 87%; and accuracy ranged from 74% to 82%. 13 studies evaluated C-TIRADS (20, 69, 70, 72, 80, 87, 120, 137–142), with sensitivity ranging from 70% (C-TIRADS TR4c) to 95% (C-TIRADS TR4a); specificity ranging from 54% (C-TIRADS TR4a) to 92% (C-TIRADS TR4c); and accuracy ranging from 75% (C-TIRADS TR4a) to 88% (C-TIRADS TR4b). 12 studies evaluated S-Detect (45, 46, 65, 112, 114, 133, 134, 143–147), with sensitivity ranging from 73% to 88%; specificity ranging from 66% to 86%; accuracy ranging from 72% to 83%. Heterogeneity was found to be significant across all evaluated risk category thresholds for all systems in terms of sensitivity, specificity, and accuracy (all I^2^>75%).

**Table 2:** Results of meta-analysis of diagnostic performance of individual risk stratification systems.

System and Category	Sensitivity		Specificity		Accuracy		Number of Studies
	Value	I^2^	Value	I^2^	Value	I^2^	Value
ACR TIRADS							
TR3, mildly suspicious	99[98;99]	81.1	15[9;23]	98.6	44[35;53]	99.0	21
TR4, moderately suspicious	93[91;95]	96.6	58[53;63]	97.5	69[66;72]	98.5	90
TR5, highly suspicious	71[65;76]	97.2	87[84;89]	98.3	79[77;80]	96.3	79
Eu-TIRADS							
TR3, low risk	93[86;100]	100	17[6;28]	98.0	31[20;41]	97.8	6
TR4, intermediate risk	93[89;95]	94.2	51[43;58]	98.5	62[56;68]	99.2	29
TR5, high risk	75[66;82]	96.2	82[75;87]	98.6	78[74;82]	98.1	24
C-TIRADS							
TR4a, low malignancy suspicion	94[92;95]	84.6	67[54;78]	97.4	80[75;86]	95.9	6
TR4b, moderate malignancy suspicion	92[90;94]	97.2	71[55;83]	98.74	79[70;88]	97.8	4
TR4c, extremely malignancy suspicion	75[70;78]	82.6	87[80;92]	96.1	82[77;87]	96.7	3
S-Detect							
Possible malignance	81[73;88]	87.9	77[66;86]	97.2	77[72;83]	92.6	12

Diagnostic performance measures expressed as percentage with 95% CI in parentheses.

ACR TIRADS, American College of Radiology Thyroid Imaging Reporting and Data System; Eu-TIRADS, European Thyroid Association Thyroid Imaging Reporting and Data System; C-TIRADS, Chinese version of Thyroid Imaging Reporting and Data System; S-Detect, Computer aided diagnosis system using deep learning.

### Network meta-analysis based on selected risk category thresholds for each system

**Figure 2:**
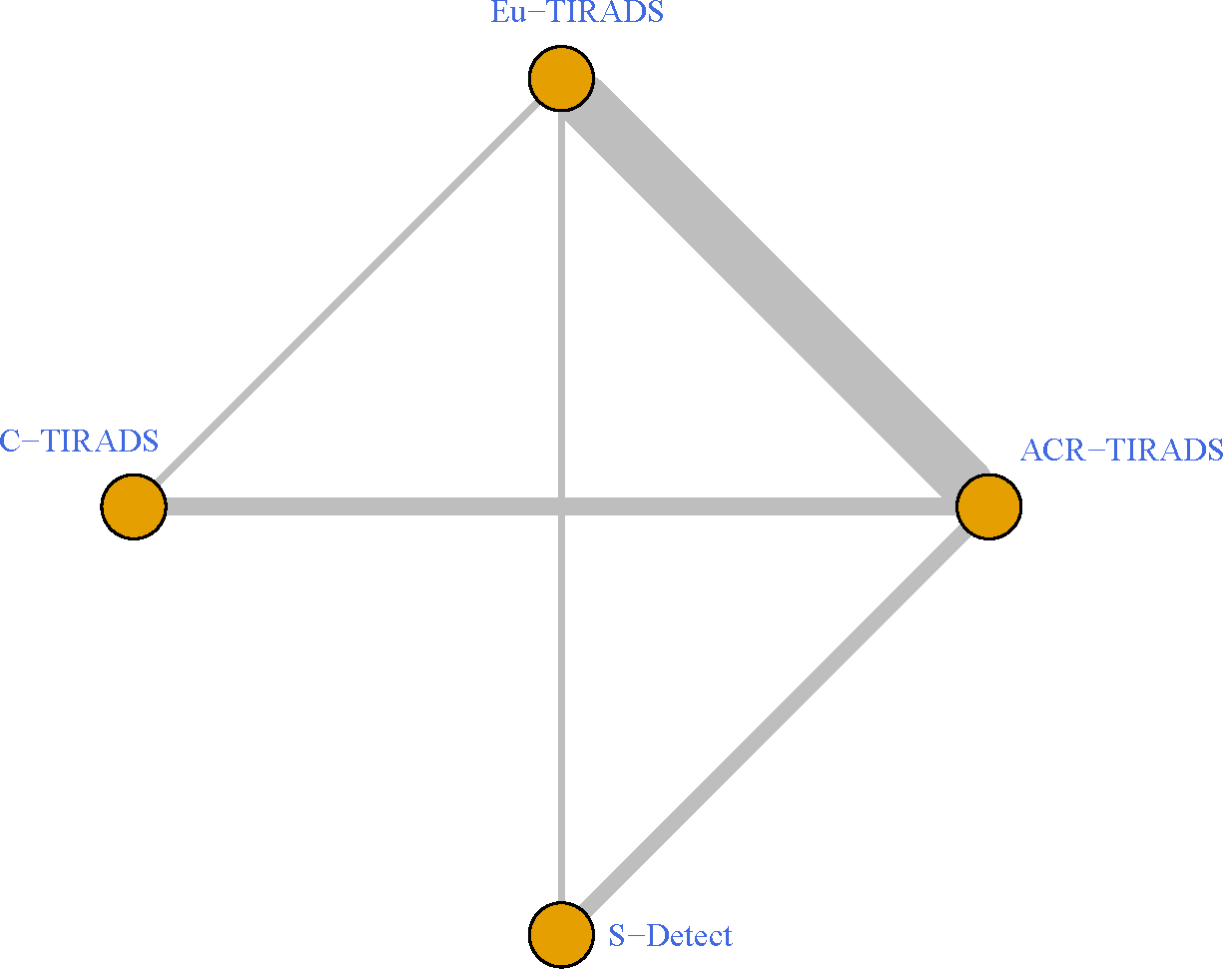
Schematic of network meta-analysis of 36 studies containing more than one risk classification system for thyroid nodules on ultrasound. Direct comparisons within individual studies were indicated by lines connecting pairs of systems. Number of studies involved in each pairwise comparison was indicated by width of lines. ACR TIRADS, American College of Radiology Thyroid Imaging Reporting and Data System; Eu-TIRADS, European Thyroid Association Thyroid Imaging Reporting and Data System; C-TIRADS, Chinese version of Thyroid Imaging Reporting and Data System; S-Detect: Computer aided diagnosis system using deep learning.

**Figure 3:**
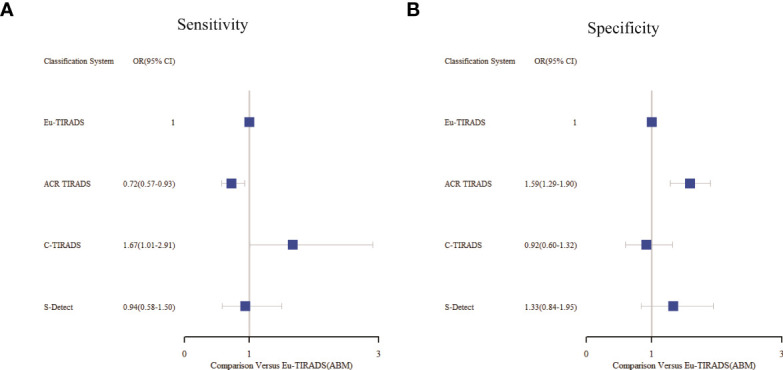
Summary of sensitivity **(A)** and specificity **(B)** from network meta-analysis among the best cut-offs in each system. Tick marks indicate ORs, blue boxes around tick marks are proportional to precision of estimates, and horizontal lines indicate 95% CIs. Eu-TIRADS TR5 served as reference for ORs. ACR TIRADS, American College of Radiology Thyroid Imaging Reporting and Data System; Eu-TIRADS, European Thyroid Association Thyroid Imaging Reporting and Data System; C-TIRADS, Chinese version of Thyroid Imaging Reporting and Data System; S-Detect, Computer aided diagnosis system using deep learning; ABM, Arm-based model; OR, Odds ratio.

As displayed in **Table 3**, the network meta-analysis was conducted on threshold categories with the highest accuracy, specifically TR5 for Eu-TIRADS, TR5 for ACR TIRADS, and possible malignancy for S-Detect. Owing to similar accuracy performance among TR4a, TR4b, and TR4c, these threshold categories of C-TIRADS were respectively compared with Eu-TIRADS TR5, ACR TIRADS TR5, and S-Detect possible malignancy. Finally, a total of 88 studies including 59,304 nodules were included in network meta-analysis based on the best cut-off category in each system. **Figure 2** depicted the direct comparisons within the network meta-analysis, where the width of the lines between systems represented the number of studies included in each comparison. Eu-TIRADS was chosen as reference system for network meta-analysis, given that an appropriate number of studies can partially ensure results’ reliability. **Figure 3** graphically displayed the results of the network meta-analysis in terms of the OR for comparisons of sensitivity, specificity, and accuracy of other systems with Eu-TIRADS.

**Table 3:** Comparisons of sensitivity, specificity, and DOR of risk classification systems, based on network meta-analysis.

System	Cut-off	Sensitivity			Specificity			DOR
		%	RR	OR	%	RR	OR	Value
Eu-TIRADS	TR5	0.73	1.00	1.00	0.75	1.00	1.00	8.19
ACR TIRADS	TR5	0.67	0.92	0.76	0.82	1.09	1.52	9.27
C-TIRADS	TR4a	0.96	1.32	10.40	0.38	0.50	0.21	16.44
S-Detect	Possibly malignant	0.73	1.01	1.07	0.78	1.04	1.20	9.98
/								
Eu-TIRADS	TR5	0.75	1.00	1.00	0.73	1.00	1.00	8.22
ACR TIRADS	TR5	0.68	0.91	0.72	0.81	1.11	1.59	9.20
C-TIRADS	**TR4b**	0.82	1.10	**1.67**	0.71	0.97	0.92	**12.36**
S-Detect	Possibly malignant	0.73	0.97	0.94	0.78	1.06	1.33	9.87
/								
Eu-TIRADS	TR5	0.74	1.00	1.00	0.73	1.00	1.00	8.11
ACR TIRADS	TR5	0.68	0.91	0.74	0.81	1.11	1.57	9.18
C-TIRADS	TR4c	0.51	0.69	0.39	0.90	1.22	3.31	10.02
S-Detect	Possibly malignant	0.73	0.99	0.99	0.78	1.06	1.29	10.02

“%” represent percentages.

ACR TIRADS, American College of Radiology Thyroid Imaging Reporting and Data System; Eu-TIRADS, European Thyroid Association Thyroid Imaging Reporting and Data System; C-TIRADS, Chinese version of Thyroid Imaging Reporting and Data System; S-Detect: Computer aided diagnosis system using deep learning; RR, Risk ratio; OR, Odds ratio; DOR, Diagnostic odds ratio.

The bold values means the optimal results of C-TIRADS TR4b were emphasized.

**Figure 4:**
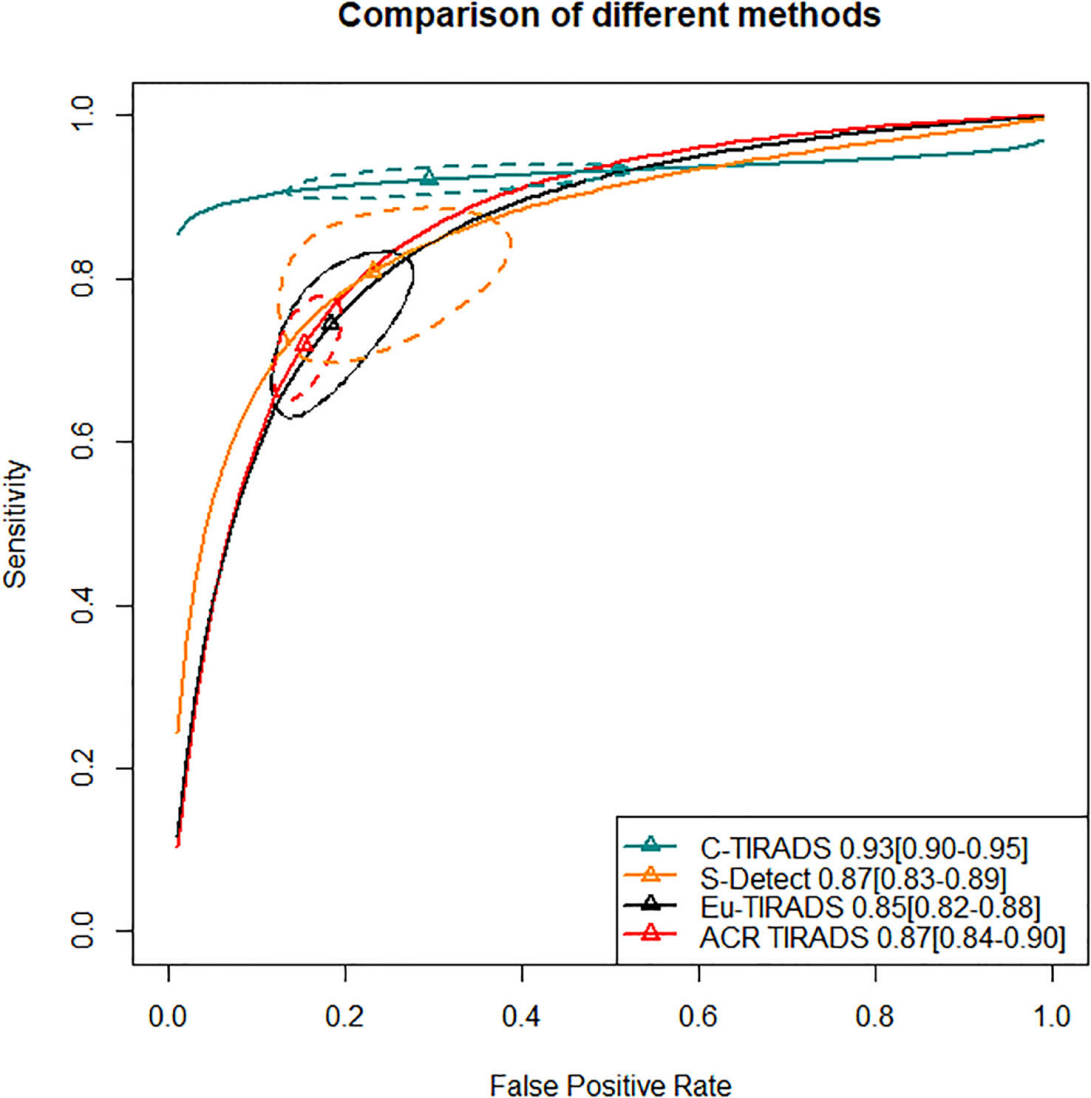
Significant separation of combined effect size point estimate of AUC of C-TIRADS TR4b, Eu-TIRADS TR5, ACR TIRADS TR5 and S-Detect possible malignancy. Additionally, the confidence interval of C-TIRADS did not overlap with those of the other diagnostic systems, indicating that C-TIRADS had the highest AUC in diagnosing the benign or malignant nature of thyroid nodules. ACR TIRADS, American College of Radiology Thyroid Imaging Reporting and Data System; Eu-TIRADS, European Thyroid Association Thyroid Imaging Reporting and Data System; C-TIRADS, Chinese version of Thyroid Imaging Reporting and Data System; S-Detect, Computer aided diagnosis system using deep learning; AUC, Area under curve.

In the second section, sensitivity for ACR TIRADS TR5 [RR= 0.91 (95% CI, 0.85–0.98); OR=0.72 (95% CI, 0.57–0.93)] was significantly lower than Eu-TIRADS TR5, and lower (although not significantly) for S-Detect possible malignancy [RR= 0.97 (95% CI, 0.85–1.10); OR=0.94 (95% CI, 0.58–1.50)]. In contrast, the sensitivity of C-TIRADS [RR= 1.10 (95% CI, 1.00–1.22); OR=1.67 (95% CI, 1.01–2.91)] was significantly higher than Eu-TIRADS. The specificity was significantly higher than Eu-TIRADS for ACR TIRADS [RR= 1.11 (95% CI, 1.05–1.17); OR=1.59 (95% CI, 1.29–1.90)], and higher (although not significantly) for S-Detect [RR= 1.06 (95% CI, 0.95–1.17); OR=1.33 (95% CI, 0.84–1.95). On the other hand, the specificity was lower (although not significantly) than Eu-TIRADS for C-TIRADS [RR= 0.97 (95% CI, 0.86–1.07); OR=0.92 (95% CI, 0.60–1.32)]. DOR is commonly applied in systematic meta-analysis to comprehensively assess the performance of diagnostic tests in different studies by combining relationship between true positive rate and false positive rate. DOR close to 1 indicates less accuracy in the test. The DOR of C-TIRADS [12.36 (95% CI, 6.55–22.24)] ranked first, followed in order by S-Detect [9.87 (95% CI, 6.09–15.46)], ACR TIRADS [9.20 (95% CI, 7.58–10.97)], and Eu-TIRADS [8.22 (95% CI, 6.05–10.82)]. **Figure 4** presented the results of SROC analysis among four risk stratification systems. It was found that C-TIRADS 4b had significantly higher AUC [0.93 (95% CI, 0.90–0.95)] compared to the other systems.

### Subgroup analysis

In network meta-analysis, within-group heterogeneity and between-group heterogeneity were high (all I^2^>75%, p<0.1). Subgroup analysis found that publication year, age, gender, and race were not contributors to the heterogeneity (all I^2^>75%, p<0.1).

### Publication bias

**Figure 5:**
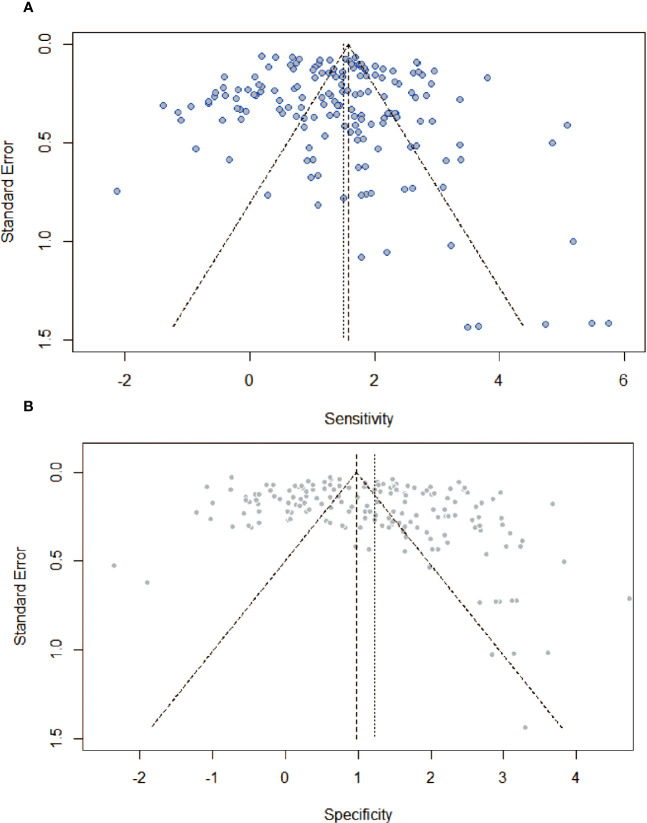
Assessment of publication bias by funnel plots with respect to **(A)** sensitivity, and **(B)** specificity. There was no providing evidence of publication bias.

**Figure 5** showed funnel plots of sensitivity and specificity. Visually, each point was evenly distributed on both sides. There were a few individual points with significant deviation, but they had minimal impact on the overall results. From the funnel plot, there was no obvious publication bias.

## Discussion

This study took on network meta-analysis of four ultrasound-based risk stratification systems to compare their diagnostic performance in thyroid malignancy. It deemed that the highest accuracy threshold categories as TR5 for Eu-TIRADS, TR5 for ACR TIRADS, and possible malignancy for S-Detect. In the network meta-analysis respectively with C-TIRADS TR4a/4b/4c, the best threshold category of C-TIRADS was identified as TR4b. At the optimal category thresholds, the sensitivity and specificity of the risk stratification options ranged from 68–82% and 71–81%, respectively. Based on AUC determined for these category thresholds, SROC analysis also revealed the best diagnostic test performance for C-TIRADS TR4b.

Ultrasound is taken as the best imaging modality for determining malignant risk of thyroid nodules (125). However, in China, 82.3% of thyroid surgeons or endocrinologists express their concern regarding the overuse of surgery (22). However, each risk stratification system was proposed according to different racial populations and would vary in their assignment of risk categories to one nodule (37). Thus, in view of large population in China, it is necessary to explore system with relatively satisfactory diagnostic performance, consequently reducing medical care burden (45). In addition, acknowledging different diagnostic performances in various ultrasound-based risk stratification systems and combining their unique strengths will enable clinicians to make evidence-based choices about the most appropriate system for personal practice. Moreover, consistency and standardization of thyroid nodule evaluation across different healthcare settings will aid in muti-center validation of clinical trials (133).

Both C-TIRADS and ACR TIRADS are score-based systems that employ certain scores and weights to assess likelihood of nodule malignancy (22, 72). In contrast, Eu-TIRADS is a pattern-based system (126). On the other hand, S-Detect is a radiomics-based system that is driven by deep learning algorithms to automatically classify and evaluate thyroid nodules (112). Relative to Eu-TIRADS and C-TIRADS, ACR TIRADS has highly comprehensive and detailed reference entry at TR5 cutoff, which might lead to an increase in diagnostic specificity but decrease in sensitivity (51). C-TIRADS with higher sensitivity, diagnostic accuracy, and DOR than other systems might be due to more reasonable feature selection for malignancy diagnosis by TR4 subdivision. Based on the outcomes of multiple logistic regression and counting analysis, solid composition, microcalcification, marked hypoechoic features, blurred margins, irregular margins or extrathyroidal extension, and vertical orientation are considered suspicious malignant ultrasound features in C-TIRADS, while comet tail artifact is a benign feature (139). Through high-dimensional imaging features in ultrasound, S-Detect demonstrated a relatively high level of specificity, which was consistent with previous research findings (145). In addition, S-Detect, whose DOR ranked second among risk stratification systems, exhibited superior diagnostic performance, providing compelling support for its clinical utility (146).

There were some limitations in this study. Firstly, this network meta-analysis took histologic or cytologic tests as reference standards, and its results might not be extrapolated to other situations, like only clinical follow-up. Secondly, diagnostic performance might be affected by uncontrolled variables, such as scan technique, ultrasound equipment quality, and interpreter experience. The inclusion of studies conducted in various countries worldwide, with diverse populations, reference standards, and trial designs, may introduce some degree of uncertainty into direct/indirect comparisons. Thirdly, literature number of C-TIRADS and S-Detect was relatively small, which might introduce publication bias. Hence, this systemic review was a preliminary study to discuss C-TIRADS applicability (53). Fourthly, another limitation of the study is incomplete collection of clinical data, such as nodule size (≥10mm and <10mm) and age stratification, which may contribute to unobserved heterogeneity and limit the generalizability of results (16). Up to now, almost all literature focused on middle-aged and elderly populations, except several studies on children (81, 82, 114, 124), emphasizing more attention should be paid to pediatric patients in the future. Finally, it ought to be noted that comparisons of the systems were based on a single meta-analysis-derived best cutoff. It is important to recognize that our selected threshold per system might not fully reflect real-world experience in applying these systems with a range of risk categories. Interestingly, there is a 5% chance of malignancy at C-TIRADS TR4a in Chinese (137), which will require intimate monitoring or even puncture biopsy. How to balance the applicability between C-TIRADS TR4a and TR4b is of significance to avoid unnecessary invasive biopsy.

## Conclusion

This network meta-analysis evaluated four risk stratification options on ultrasound for thyroid nodules. Sensitivity, DOC, and accuracy were the highest for C-TIRADS TR4b (moderate malignancy suspicion); sensitivity was lowest but specificity was highest for ACR TIRADS TR5 (highly suspicious). This tentative assessment of risk stratification systems for thyroid nodules may assist in future system updates and guide decisions regarding system implementation.

## Data availability statement

The raw data supporting the conclusions of this article will be made available by the authors, without undue reservation.

## Author contributions

JL and LTY were responsible for the review concept and design. LTY, CL, ZC and SQH drafted the manuscript. JL and LTY provided critical revision of the manuscript for important intellectual content. All authors contributed to the article and approved the submitted version.
