# Supplementary figures and images for: Diagnostic efficiency among Eu-/C-/ACR-TIRADS and S-Detect for thyroid nodules: a systematic review and network meta-analysis
Source: Front Endocrinol (Lausanne). 2023 Aug 31;14:1227339. doi: 10.3389/fendo.2023.1227339 (PMC10501732; doi:10.3389/fendo.2023.1227339)

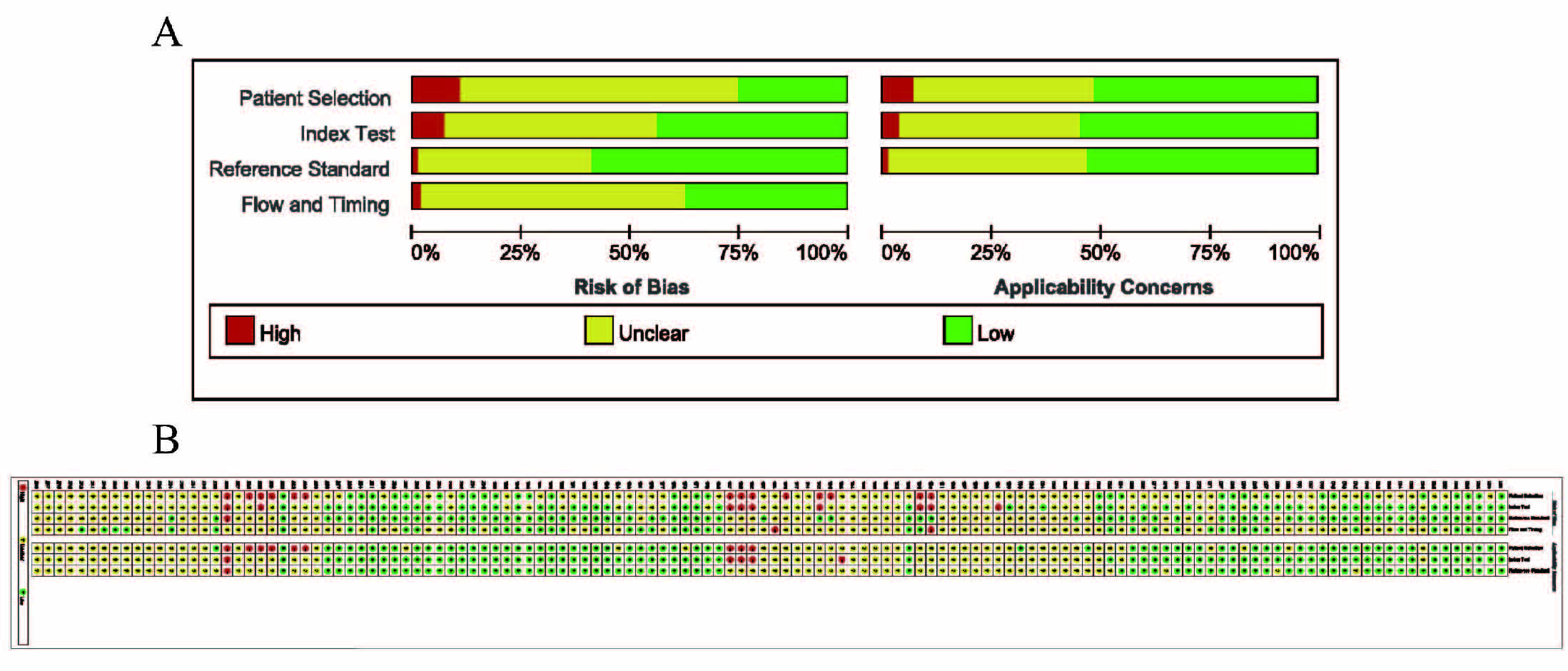

Supplement: Supplementary file 1 [file Image_1.jpeg]
